# Circulating tumor DNA is detectable in canine histiocytic sarcoma, oral malignant melanoma, and multicentric lymphoma

**DOI:** 10.1038/s41598-020-80332-y

**Published:** 2021-01-13

**Authors:** Anaïs Prouteau, Jérôme Alexandre Denis, Pauline De Fornel, Edouard Cadieu, Thomas Derrien, Camille Kergal, Nadine Botherel, Ronan Ulvé, Mélanie Rault, Amira Bouzidi, Romain François, Laetitia Dorso, Alexandra Lespagnol, Patrick Devauchelle, Jérôme Abadie, Catherine André, Benoît Hédan

**Affiliations:** 1 Univ Rennes, CNRS, IGDR (Institut de génétique et développement de Rennes) UMR6290, 35000 Rennes, France; 2 Sorbonne University, Paris, France; 3 INSERM UMR_S 938, Endocrinology and Oncology Biochemistry Department, APHP Pitié-Salpêtrière Hospital, Paris, France; 4 Micen Vet, Créteil, France; 5 Department of Biology, Pathology and Food Sciences, Oniris, Laboniris, Nantes, France; 6 Laboratory of Somatic Genetic of Cancers, Hospital of Rennes, Rennes, France

**Keywords:** Cancer genetics, Cancer models, Haematological cancer, Sarcoma, Tumour biomarkers

## Abstract

Circulating tumor DNA (ctDNA) has become an attractive biomarker in human oncology, and its use may be informative in canine cancer. Thus, we used droplet digital PCR or PCR for antigen receptor rearrangement, to explore tumor-specific point mutations, copy number alterations, and chromosomal rearrangements in the plasma of cancer-affected dogs. We detected ctDNA in 21/23 (91.3%) of histiocytic sarcoma (HS), 2/8 (25%) of oral melanoma, and 12/13 (92.3%) of lymphoma cases. The utility of ctDNA in diagnosing HS was explored in 133 dogs, including 49 with HS, and the screening of recurrent *PTPN11* mutations in plasma had a specificity of 98.8% and a sensitivity between 42.8 and 77% according to the clinical presentation of HS. Sensitivity was greater in visceral forms and especially related to pulmonary location. Follow-up of four dogs by targeting lymphoma-specific antigen receptor rearrangement in plasma showed that minimal residual disease detection was concordant with clinical evaluation and treatment response. Thus, our study shows that ctDNA is detectable in the plasma of cancer-affected dogs and is a promising biomarker for diagnosis and clinical follow-up. ctDNA detection appears to be useful in comparative oncology research due to growing interest in the study of natural canine tumors and exploration of new therapies.

## Introduction

Cell-free DNA (cfDNA) circulates in the plasma and other body fluids, such as urine, cerebrospinal fluid, pleural fluid, and saliva, as short double-stranded DNA fragments of approximately 160 base pairs. In healthy individuals, cfDNA is released during cell death that occurs via apoptosis or necrosis or by active secretion. It is mainly derived from the hematopoietic lineage with minimal contribution from other tissues^1–4^. Since its first discovery in humans in 1948^5^, the presence of cfDNA in the blood has been a known indicator of various pathological processes, such as inflammatory disease, sepsis, trauma, stroke, and cancer, and its concentration is correlated with the severity of disease and prognosis^2,6–8^. In patients with cancer, cfDNA is partially derived from tumor cells and is referred to as circulating tumor DNA (ctDNA). ctDNA carries tumor-related genetic and epigenetic alterations that are relevant to the development, progression, and resistance to therapy of cancer^8^.

Next-generation sequencing (NGS) has been used in human medicine to identify genetic alterations in ctDNA, which may include mutations in tumor suppressor genes such as *TP53* in patients with head and neck cancer^9^, mutations in oncogenes such as *EGFR* or *KRAS* in patients with non-small-cell lung cancer^10,11^, and copy number alterations (CNA) and chromosomal rearrangements as observed in the variable-diversity-joining (VDJ) receptor gene sequences in lymphoma^12^. In the past few decades, strategies including the study of loss of heterozygosity (LOH), DNA methylation, DNA integrity, NGS, or digital PCR have been successfully developed to detect somatic alterations in the plasma of patients, making ctDNA a new powerful biomarker for cancer^8,13^. In addition to being a minimally invasive and robust approach, the analysis of ctDNA has several clinical applications, such as the early detection of cancer, prognosis, real-time monitoring of treatment response, and the identification of appropriate therapeutic targets and resistance mechanisms^2,8,10,11,14^.

In veterinary medicine, the use of cfDNA as a biomarker has recently gained attention, particularly in dogs. As in humans, it has been found that cfDNA has a short half-life in canine plasma (approximately 5 h)^15^, and various diseases cause an increase in its concentration, including immune-mediated hemolytic anemia^16^, sepsis, severe trauma, and inflammation^17–19^. Moreover, the concentration of cfDNA is correlated with the severity of various diseases and prognosis in dogs^18^ and is of potential interest in canine cancers. Studies have shown that dogs with lymphoid neoplasia and mammary carcinoma have higher plasma cfDNA concentrations than do controls^19,20^. In the last decade, the presence of recurrent somatic alterations has been identified in several canine cancers, such as multicentric lymphoma^21,22^, histiocytic sarcoma (HS)^23–27^, and oral malignant melanoma (OMM)^28–32^. The detection of cancer-specific recurrent somatic alterations in plasma may allow the development of novel minimally invasive biomarkers for the diagnosis, prognosis, and assessment of responses to treatment in veterinary medicine. Additionally, tests based on ctDNA detection may be useful in the field of comparative oncology research. Indeed, naturally occurring canine cancers have become relevant models for the study of rare human cancers, and have been used for the discovery of mutations for the development and screening of targeted therapies^27^.

In this proof-of-concept study, our objective was to examine the presence of ctDNA and whether several types of recurrent somatic alterations are detectable in the plasma of dogs with three types of malignancies that may serve as models for their human counterparts, i.e., histiocytic sarcoma^27^, oral malignant melanoma^33^, and multicentric lymphoma^34^. Further, we aimed to evaluate whether ctDNA can be used in veterinary medicine for cancer diagnosis and to evaluate responses to treatment with chemotherapy by monitoring minimal residual disease (MRD) in dogs with lymphoma.

## Results

### Characteristics of the cohort

#### Characteristics of the dogs

Plasma samples were collected from 49 dogs with histiocytic sarcoma (17 disseminated forms, 30 localized forms, and 2 unknown), 16 dogs with oral melanoma (OMM), and 25 dogs with multicentric lymphoma (including 18 high-grade B-cell, 2 high-grade T-cell, and 3 low-grade lymphomas) (Supplementary Table 1). Matched tumor samples were available from 45 dogs with HS, 10 with OMM, and 14 with multicentric high-grade B-cell lymphomas. Plasma samples from 19 healthy dogs (mean age at sampling was 7.4 years), 14 dogs with non-cancerous diseases (mean age at sampling was 7.3 years), and 10 dogs with various cancers (mean age at sampling was 6.9 years) were collected as controls. Among the healthy dogs and dogs with cancer, the most represented breeds were the Bernese mountain dog (9/19 and 45/100, respectively) and the flat-coated retriever (n = 8/19 and 7/100, respectively).

#### Quantification of cfDNA in the plasma

**Figure 1:**
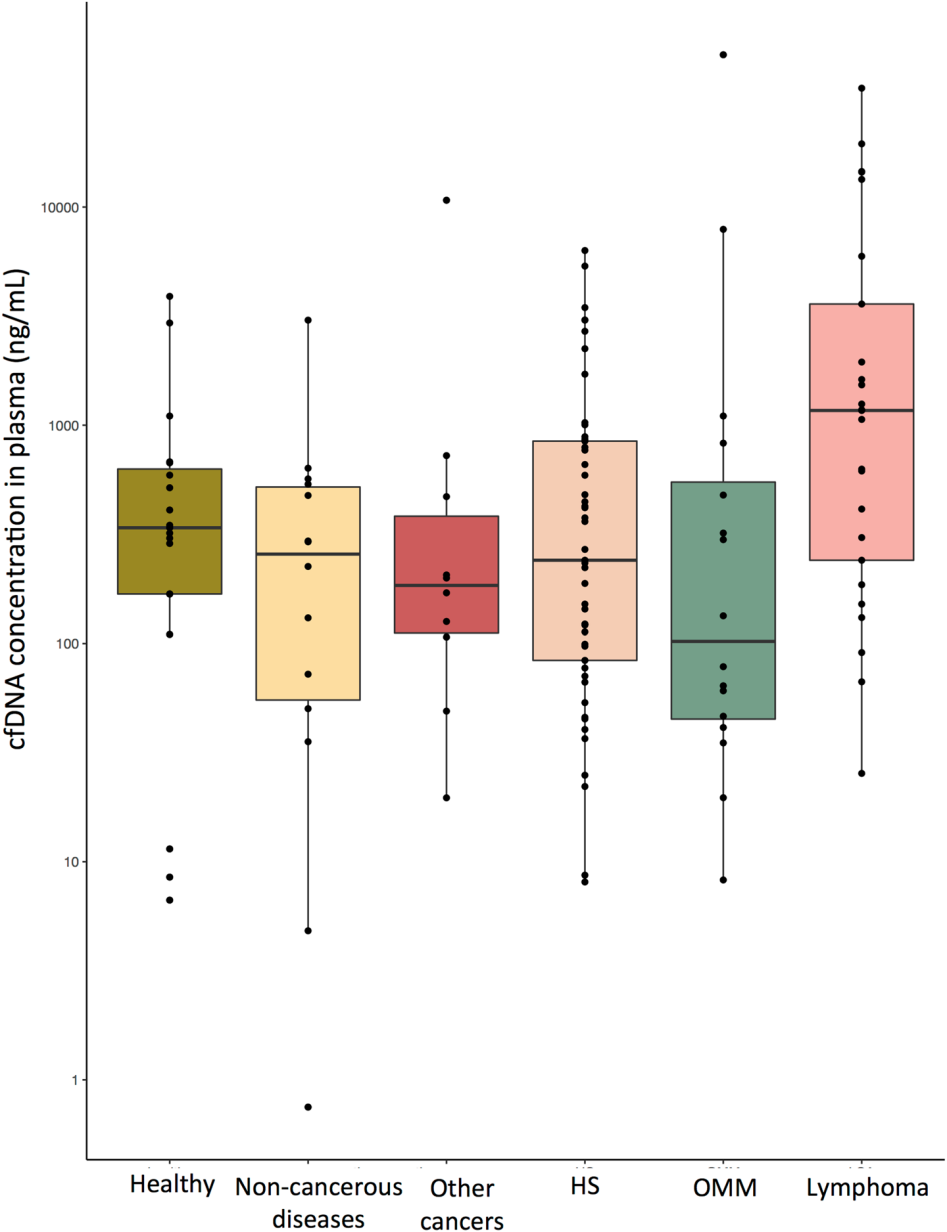
Quantification of cfDNA (ng/mL) in the plasma of dogs in different conditions: healthy, non-cancerous disease, various cancers, HS, OMM, and multicentric lymphoma (drawing produced by R studio software Version 1.1.463; Vienna, Austria^60^). The amount of DNA was determined via the Poisson distribution.

The cfDNA in the plasma of dogs was accurately quantified by the Droplet Digital PCR (ddPCR) method using the wild-type (wt) *PTPN11* gene as a reference. The median cfDNA concentrations in healthy, HS, multicentric lymphoma, OMM, other cancers, and non-cancerous dogs were 339 ng/mL (mean 678 ng/mL; range 6.7–3895 ng/mL), 240 ng/mL (mean 827 ng/mL; range 8.1–6320 ng/mL), 1168 ng/mL (mean 4764 ng/mL; range 25.4–35,079 ng/mL), 106 ng/mL (mean 3833 ng/mL; range 8.2–49,910 ng/mL), 185 ng/mL (mean 1282 ng/mL; range 19.6–10,746 ng/mL), and 259 ng/mL (mean 454 ng/mL; range 0.7–3030 ng/mL), respectively (Fig. 1). Although dogs with multicentric lymphoma had higher concentrations of cfDNA in the plasma than the other groups, this was not significant (Kruskal–Wallis test, *p* = 0.53). Moreover, advanced clinical stage was not associated with higher quantities of cfDNA in our cohort of dogs with lymphomas (stage V vs. III–IV; Wilcoxon test, *p* = 0.45) or oral melanomas (stage IV vs. II–III; Wilcoxon test, *p* = 0.76). Similarly, the clinical presentation of HS was not linked to the quantity of cfDNA in the plasma (disseminated vs. localized forms, Wilcoxon test, *p* = 0.26; external vs. visceral forms, Wilcoxon test, *p* = 0.39).

Taken together, these findings indicate that cfDNA is not a sensitive marker for tumor diagnosis and that the detection of tumor-specific genetic alterations is required to confirm the existence of a malignancy.

### Detection of ctDNA in the plasma of dogs with cancer

#### Detection of ctDNA in canine histiocytic sarcoma by targeting tumor-specific mutations

The knowledge of tumor-specific and recurrent mutation hotspots is required to detect ctDNA by the screening of point mutations. *PTPN11* is an oncogene involved in the MAPKinase pathway and is mutated in 23–56.7% of HS cases, mainly in two hotspots (E76K or G503V), depending on clinical presentation^25–27^.

**Figure 2:**
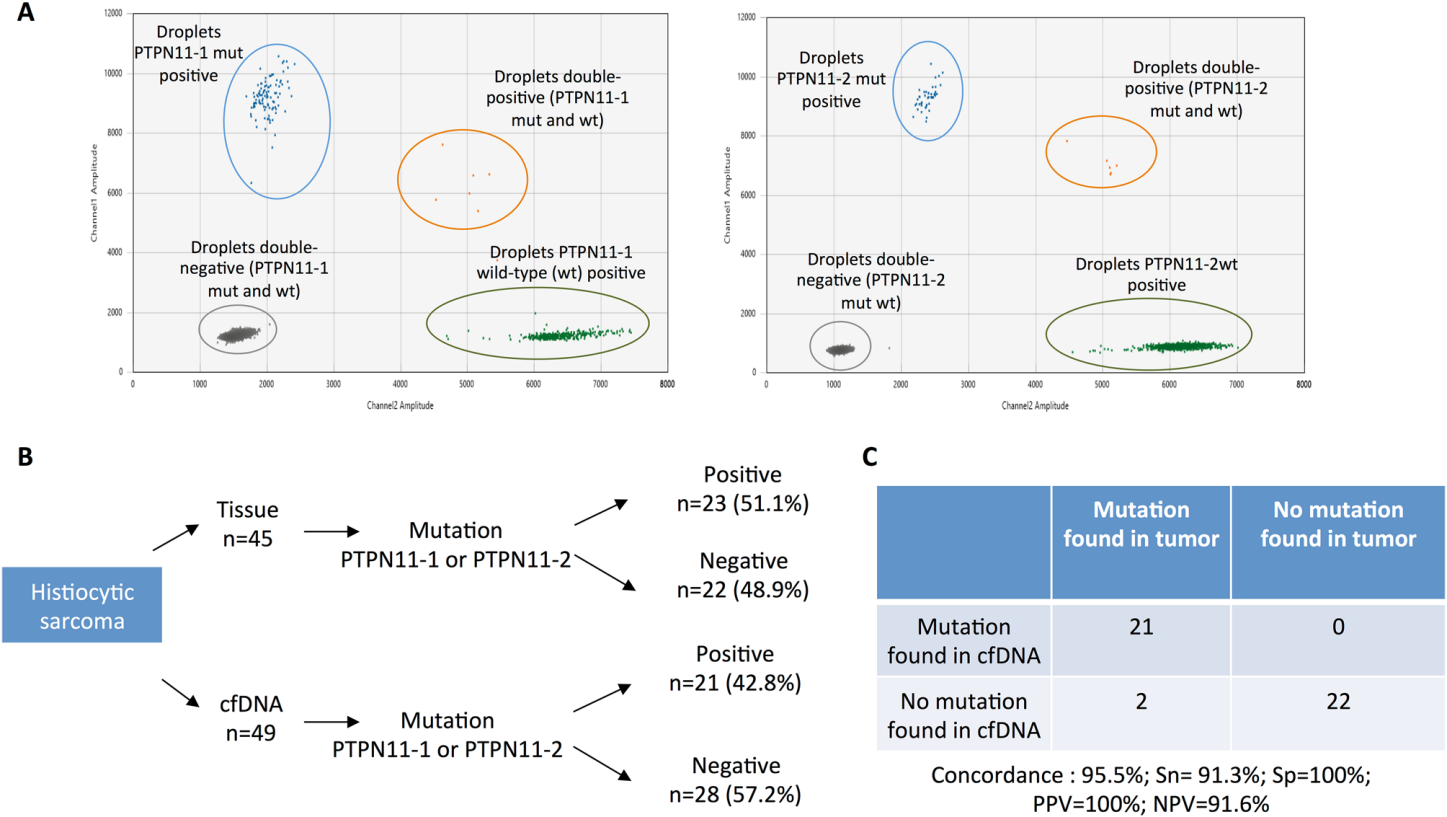
(**A**) An example of data output from the ddPCR multiplex assay for absolute quantification of *PTPN11* mutations (PTPN11-1 for E76K variant and PTPN11-2 for the G503V variant) in canine HS. Exemplary two-dimensional cluster plots from QuantaSoft software (version 1.7.4, Bio-Rad, freely available after registration: https://www.bio-rad.com/fr-fr/sku/1864011-quantasoft-software-regulatory-edition) in which PTPN11-1mut (left) or PTPN11-2mut (right) are plotted against the PTPN11-1 wild-type (wt) and PTPN11-2wt respectively. The droplets form clusters that should arrange orthogonally to each other and represent the *PTPN11*mut/*PTPN11*wt negative (double-negative droplets, grey), *PTPN11*mut positive (blue), *PTPN11*wt positive (green), and *PTPN11*mut/*PTPN1*1wt positive samples (double-positive droplets, orange). (**B**) Flowchart summarizing the results of the ddPCR assay targeting the *PTPN11* mutations (E76K and G503V) in the tumor and plasma samples from 49 dogs with HS. cfDNA: cell-free DNA. (**C**) Parameters of the test for the detection of the *PTPN11* mutation in the plasma of dogs with HS. *PPV* positive predictive value, *NPV* negative predictive value.

Our results showed that *PTPN11* mutations were found in 23/45 (51.1%) of HS tumor samples. Among these 23 dogs, the same mutation was found in the plasma of 21/23 (91.3%). Further, for the 21 plasma samples with a detectable *PTPN11* mutation, the variant allelic fraction ranged from 0.056–36% (Supplementary Table 1). No mutations were detected in the plasma of dogs with wt *PTPN11* tumors (Fig. 2).

#### Detection of ctDNA in canines with OMM by targeting copy number alterations (CNAs)

To detect ctDNA in OMM patients, we targeted the *MDM2* and *TRPM7* amplifications that are present in up to 50% of canines with OMM^28,30–32^. The copy number was determined in tumor and plasma samples via ddPCR. Of the ten OMM cases for which tumor samples were available, *MDM2* amplification was found in 3/10 tumors and was detected in the plasma of 1/3 of the corresponding dogs (Table 1). Similarly, *TRPM7* amplification was found in 8/10 tumors, but was detected in the plasma of only one dog (Table 1). These results indicate that specific tumor CNAs were detectable in the plasma of 2/8 (25%) dogs with OMM.

**Table 1:** Results of the ddPCR assay to identify the CNA of the *TRPM7* and *MDM2* genes in tumors and plasma of dogs with OMM.

Dog Id	Tumor	cfDNA
	Gain detected: copy number (95% confidence interval)	Gain detected: copy number (95% confidence interval)
19006	*TRPM7*: 37.90 (28.41–47.39)	*TRPM7*: 2.80 (2.58–3.03)
18394	*TRPM7*: 7.98 (7.71–8.25)	No
18657	*TRPM7*: 3.01 (2.89–3.14)	No
18718	*MDM2*: 21.26 (19.37–23.15)	*MDM2*: 3.02 (2.19–3.85)
	*TRPM7*: 7.46 (7.17–7.75)	No
18395	*MDM2*: 52.96 (45.74–60.17)	No
	*TRPM7*: 8.78 (8.37–9.19)	No
18303	*TRPM7*: 8.88 (8.55–9.20)	No
18333	*MDM2*: 4.30 (3.91–4.68)	No
	*TRPM7*: 27.24 (25.94–28.54)	No
18848	*TRPM7*: 9.12 (8.78–9.46)	No

Eight dogs had at least one gene amplified in their tumors, and two dogs had a copy number imbalance in the plasma.

#### Detection of ctDNA in canine multicentric lymphoma by targeting chromosomal rearrangements

**Figure 3:**
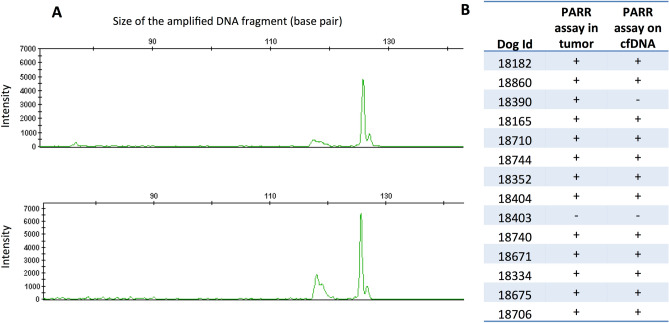
(**A**) Results of a PARR genotyping on the DNA extracted from a tumor sample (above) and plasma (below) from a dog with high-grade multicentric lymphoma (Id 18175) (drawing produced by GeneMapper Software 4.1: https://www.thermofisher.com/ order/catalog/product/4366925). The same clonal antigen receptor rearrangement (IgH major) was found with a high intensity in the tumor and the plasma. (**B**) Results of the PARR assay on canine lymphoma. The results on DNA extracted from the tumor and DNA extracted from plasma are shown. PARR was positive (clonal band) in the tumor and cfDNA for 12/13 dogs.

ctDNA can be identified by tumor-specific chromosomal rearrangements, as is performed for humans with multicentric lymphomas^12^. VDJ recombination of the genes for immunoglobulins and T-cell receptors occurs in the early stages of lymphocyte maturation and is specific to the lymphocyte clone. Thus, in lymphomas, the VDJ sequence is unique and constitutes a specific marker of the tumor clone^12^. To identify lymphoma-specific VDJ rearrangements, we performed PCRs for antigen receptor rearrangement (PARR) in 14 dogs with high-grade B-cell multicentric lymphoma (11 cases were classified as diffuse large B-cell lymphomas, and 3 were not classified). PARR was positive and detected a clonal rearrangement in the tumors from 13/14 dogs. To detect ctDNA, PARR was performed on cfDNA extracted from plasma at the time of diagnosis. All samples except one were positive and had the same clone in the tumor and plasma samples (Fig. 3). These results indicate that tumor-specific chromosomal rearrangements are detectable in the plasma of dogs with multicentric lymphomas and that a majority of canine lymphoma patients (92.3% in our cohort) had detectable ctDNA at the time of diagnosis.

### The potential clinical applications of the ctDNA biomarker in veterinary medicine and oncology research

ctDNA has become an attractive biomarker for monitoring human cancer patients, with clinical applications in early cancer detection, prognosis, real-time monitoring of treatment response, and identification of appropriate therapeutic targets and resistance mechanisms, among other things. At the same time, dogs are recognized as underutilized models for developing new therapies in high-grade B-cell lymphoma^34,35^ or rare human cancers such as HS^27^. Here, we illustrate two potential applications for ctDNA detection in veterinary medicine and comparative oncology for the diagnosis and monitoring of MRD.

#### The use of ctDNA in diagnostics: *PTPN11* mutation in canine HS

The knowledge of a recurrent tumor-specific mutation may be useful in the development of non-invasive molecular diagnostic tools^36,37^. To assess the sensitivity and specificity of the *PTPN11* mutation in the screening of plasma to diagnose HS, we tested the plasma of 49 dogs with HS, 19 healthy dogs, 14 dogs with non-cancerous diseases, and 51 dogs with other cancers (multicentric lymphoma, OMM, soft tissue sarcoma, Langerhans cell sarcoma, nephroblastoma, hemangiosarcoma, and ocular melanoma) (Supplementary Table 1). The *PTPN11* mutation was detected in the plasma of 21/49 dogs with HS and 1 dog with a non-cancerous disease. The mutation was absent from the plasma of healthy dogs and those with other cancers. The screening for *PTPN11* mutations in the plasma had a specificity of 98.8% and a sensitivity of 42.8% for the diagnosis of canine HS. However, the sensitivity varied according to the characteristics of HS and its clinical presentation. It was higher among the Bernese mountain dogs (53.8%; Fisher exact test *p* = 0.0026), visceral forms of HS (59.4%; Fisher exact test, *p* = 0.0042), and in the thoracic location (77%; Fisher exact test *p* = 0.01) (Supplementary Table 2). This was due to the higher frequency of *PTPN11* mutations in Bernese mountain dogs (Fisher exact test, *p* = 0.0015), the visceral forms of HS (Fisher exact test, *p* = 0.008), and the presence of pulmonary involvement (Fisher exact test, *p* = 0.002) in this cohort.

#### The use of ctDNA for the monitoring of MRD: example of multicentric diffuse large B-cell lymphoma

**Figure 4:**
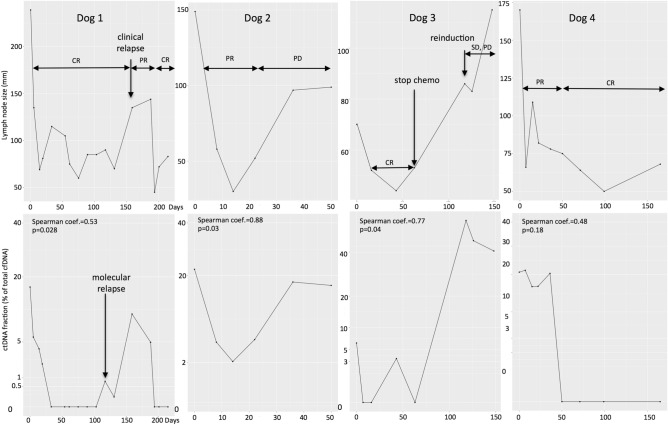
Course of disease for four dogs with lymphoma that were treated using chemotherapy (dog 1: 18182, dog 2: 18710, dog 3: 18680, dog 4: 18744) (drawing produced by R studio software Version 1.1.463; Vienna, Austria^60^). For each dog, the figures above represent the tumor burden with lymph node size (sum of longest diameter of at least 2 lymph nodes; in mm). The figures below represent the ctDNA measurement (% of total cfDNA in plasma) during the disease. For each case, a Spearman correlation analysis was performed to assess the correlation between the tumor burden and ctDNA quantity (coefficient and *p* value). *CR* complete response, *PR* partial response, *SD* stable disease, *PD* progressive disease.

In lymphoma, the unique VDJ rearrangement of the tumor clone constitutes a “barcode” that is used for the follow-up of human lymphoma patients in clinical trials^12^. In this study, four dogs with multicentric diffuse large B-cell lymphoma were included prospectively, and their lymphoma-specific VDJ rearrangements were sequenced to perform ctDNA analysis during follow-up. Three dogs received cyclophosphamide, doxorubicin, vincristine, and prednisolone (CHOP)-based chemotherapy^38–40^, and one dog (dog 3) received L-asparaginase at induction of chemotherapy, followed by vincristine injections alone. In the absence of response or relapse, L-asparaginase and lomustine were used as rescue treatments. Before the initiation of chemotherapy, the ratio of ctDNA (% of cfDNA) was determined in all dogs (mean proportion: 15.5% of total cfDNA before the initiation of chemotherapy; min = 6.25%, max = 22%). The monitoring details of each case are summarized in Supplementary File 1 and represented in Fig. 4.

In all dogs examined, the quantity of ctDNA decreased dramatically with the initiation of chemotherapy and followed the clinical response, reflecting the chemosensitivity of canine high-grade lymphoma. The complete response observed in three dogs (dogs 1, 3, and 4) matched molecular remission. Similarly, the progressive disease observed in three dogs (dogs 1, 2, and 3) matched increases in ctDNA quantity. Clinical relapse was identified in one dog (dog 1), and the molecular relapse was detected 42 days earlier. Finally, there was a significant correlation between lymph node size and ctDNA quantity in three dogs (dogs 1, 2, and 3). Altogether, these results suggest that ctDNA kinetics during chemotherapy treatment in dogs with multicentric lymphoma correlate with lymph node size and reflect the response to treatment.

## Discussion

cfDNA is an attractive biomarker for use in human medicine, particularly in oncology, and it has been evaluated in different canine diseases^16,17,19,20,41–44^. The cfDNA concentration in dogs is correlated with disease severity and prognosis^16,17,20,44^. In our study, the small sample size may be a limiting factor in identifying a link between clinical stage and cfDNA quantity in dogs with multicentric lymphoma and OMM, and larger cohorts are needed to further explore this hypothesis. The cfDNA concentrations we identified were in agreement with those from previous studies, although previous studies have reported highly variable cfDNA concentrations ranging from a median of 4–583 ng/mL in healthy dogs. This inter-study variability in the results for healthy dogs may arise due to the use of different extraction kits and quantification methods^16,20,42,44^ and limits the use of cfDNA as a global biomarker for cancer diagnosis.

In human oncology, current studies are focused on the analysis of DNA released from tumor cells in the plasma (ctDNA), which is much more cancer-specific. Currently, a ctDNA assay for the detection of *EGFR* mutations in patients with non-small-cell lung cancer (NSCLC) has been approved by the Food and Drug Administration, and ctDNA assays for *EGFR* in NSCLC and for *KRAS* in colorectal cancer are available for commercial use in Europe^3,4,45^. As in humans, ctDNA has been found in some canine cancers, such as lymphoma^20^, mammary carcinoma^41^, and more recently, pulmonary adenocarcinoma^43^. Further, a previous study demonstrated that detection of the *BRAF* mutation in urine is effective in the diagnosis of canine urothelial and prostatic carcinomas^46^. In our study, we focused on identifying ctDNA in plasma as a liquid biopsy. Our results showed that ctDNA was detected in canine HS, OMM, and high-grade multicentric lymphoma. Additionally, the high frequency of ctDNA detection among dogs with HS and lymphoma indicates that ctDNA may serve as a promising biomarker for these hematopoietic malignancies.

We identified ctDNAs thanks to several tumor-associated alterations and showed that point mutations, chromosomal rearrangements, and CNAs were detectable in the plasma of dogs with cancer. We used different methods to identify the targeted alterations, and these have certain advantages and drawbacks. It is important to highlight that the performance of ctDNA detection largely depends on the level of cfDNA in the patient’s plasma, the fraction of ctDNA, and the sensitivity of the method used. As a large amount of DNA is released into the plasma of dogs with lymphoma (median cfDNA of 1168 ng/mL), we found that ctDNA was detected in 92.3% (12/13) of dogs at the time of diagnosis. These results are similar to those of a previous study that found ctDNA in 78% (7/9) of dogs with multicentric lymphoma^20^ using the same method. In dogs with HS, cfDNA concentrations were much lower (median 240 ng/mL); however, the use of the highly sensitive ddPCR method enabled us to detect the *PTPN11* mutation in the plasma of 91.3% (21/23) dogs with tumors that had mutant *PTPN11.* The assay had positive and negative predictive values of 100% and 91.6%, respectively, for the detection of *PTPN11* mutations. Finally, low cfDNA levels may be an issue in the study of ctDNA in OMM. The most recurrent genetic alterations in cancer are CNAs targeting oncogenes such as *MDM2* and *TRPM7*^28,30–32^, and we identified these tumor-associated amplifications in cfDNA via ddPCR in 1/3 and 1/8 cases, respectively. The detection of CNAs in cfDNA is challenging and depends on the level of amplification of the gene in the tumor, which in turn may involve sequencing at high depths^41,47^ and is not yet cost-effective for veterinary medicine. In these conditions, the analysis of ctDNA for the targeting of tumor-specific CNA is inappropriate in canine melanoma.

Further, we evaluated the potential clinical application of ctDNA analysis in veterinary oncology. Two aspects were investigated: the value of ctDNA analysis as a tool for HS diagnosis and MRD follow-up in dogs with lymphoma. The diagnosis of canine HS may be challenging because of hidden tumor locations (e.g., lungs, mediastinum, and central nervous system) and the potential difficulty in differentiating it from reactive inflammatory disease or other malignancies^48^. Thus, we assessed the value of a ctDNA test to diagnose HS using a noninvasive method. In our study population of 133 dogs, we showed that the screening of *PTPN11* in the plasma for the diagnosis of HS was specific (98.8%) and sensitive (42.8%), with positive and negative predictive values of 95.5%, and 74.8%, respectively. We found that the *PTPN11* mutation was highly specific to HS and was not detected in the plasma of dogs with other cancers. A single diagnosis was classified as a false positive, that of a 12-year-old Bernese mountain dog sampled at the time of euthanasia in old age, and which was classified in the “non-cancerous diseases” group. However, the old dog may have been suffering from undiagnosed or early-stage HS, though a necropsy was not performed to confirm this hypothesis. Another explanation may be the accumulation of somatic variants in the healthy dog due to “clonal hematopoiesis” as is observed in humans^4^. In humans, this increases with age and is correlated with the risk of further development of hematological cancer. Such accumulation has not been investigated in dogs, and further studies are needed in predisposed breeds to look for *PTPN11* mutations in the plasma of apparently healthy dogs to determine whether clonal hematopoiesis precedes HS development. The relatively low sensitivity of this test (42.8%) is because *PTPN11* is not mutated in all HS cases (e.g., only 50% of cases in this study). Those mutations are especially found in Bernese mountain dogs^25^ and the visceral/disseminated forms^27^. In our study, all cases with *PTPN11* mutations were found in Bernese mountain dogs. Moreover, we targeted only the two most frequent *PTPN11* mutations (E76K and G503V), and some HS cases may have other *PTPN11* mutations^27,49^. Recently, mutations in *KRAS* and *BRAF* were found in 3–7% and less than 1%, respectively, of canine HS cases and were found to be exclusive to the *PTPN11* mutation^25,27^. Thus, the sensitivity of this diagnostic test can be improved by integrating the other mutations for *PTPN11*, *KRAS*, and *BRAF* in the ddPCR assay. The best sensitivity was obtained in the subgroup of dogs with visceral forms and pulmonary involvement (10/13, 77%). Therefore, the screening of *PTPN11* mutations in the plasma may be useful for the diagnosis of HS, especially in cases of pulmonary involvement and in Bernese mountain dogs. Further studies are needed to define the value of this test in a wider population by including more breeds and to explore the potential of this biomarker in early-stage diagnosis and follow-up treatment of dogs with HS. In the future, this test may be considered to guide treatment procedures toward targeted therapy in veterinary oncology practice. Indeed, we and others have shown that *PTPN11* mutations are associated with the activation of the MAPK pathway, and in vitro tests have shown that canine HS cell lines are sensitive to MEK inhibitors^27,49,50^. Our ctDNA assay had a sensitivity of 91.3% and a specificity of 100% for the detection of *PTPN11* mutations in the plasma of dogs carrying *PTPN11*-mutated HS, and it could efficiently select dogs that may benefit from this targeted therapy. Finally, as the same mutations for *PTPN11* are found in disseminated human HS^27^, the identification and kinetic quantification of such driver mutations in the plasma are key to proposing and evaluating new targeted therapies in canine models for rare and aggressive human cancer, with benefits for both veterinary and human medicine.

Another promising application of ctDNA analysis in veterinary medicine is the follow-up of MRD during chemotherapy to assess treatment efficacy. In our pilot study, we found that ctDNA levels were correlated with the clinical evaluation of canine multicentric diffuse large B-cell lymphomas, and in the one dog that experienced a relapse under treatment, ctDNA was detected 42 days before clinical relapse. These results are concordant with the studies of MRD targeting circulating tumor cells by real-time PCR, which showed that clinical relapse could be anticipated by molecular evaluation until 86 days^51^. Further studies are needed to explore the potential of ctDNA in monitoring MRD in canine lymphomas and to demonstrate its utility in the treatment course in dogs. Nevertheless, these findings as well as the detection of ctDNA 1 year after the surgical excision of a mammary carcinoma due to lung metastasis^41^ suggest that ctDNA analysis in veterinary oncology may serve as an objective parameter for treatment response and earlier relapse detection.

In conclusion, this study shows that several somatic alterations can be found in the plasma of dogs with HS, OMM, and multicentric lymphoma, indicating the presence of ctDNA in canine cancers, especially in hematopoietic malignancies. We explored two potential applications of this minimally invasive assay. First, the HS signature of *PTPN11* mutations and their detection in plasma may be used as a ctDNA diagnostic test to confirm the occurrence of HS, especially in visceral forms. Second, the search for lymphoma-specific rearrangements in the plasma of affected dogs appears to be a promising personalized method to assess treatment efficacy and follow-up. ctDNA has numerous applications in human medicine and appears to be a promising noninvasive tool in veterinary medicine with potential for future comparative oncology studies.

## Methods

### Sample collection

Blood and tissue biopsy samples from dogs were collected by veterinarians through the Cani-DNA BRC (http://dog-genetics.genouest.org). For each dog, 2–5 mL of blood was taken during a routine examination or the standard care protocol for the dog, with written consent from the owners. Blood was collected in ethylenediaminetetraacetic acid (EDTA) or Streck tubes (Streck Cell-Free DNA BCT Genomax). Plasma was separated from the blood cells and cell debris by centrifugation at 1500 × *g* for 10 min followed by a spin of 16,000×*g* for 10 min and was stored at − 20 °C before DNA purification. The work with canine samples was approved by the CNRS ethical board, France (35-238-13), and the methods were performed per the relevant regulations. The diagnosis of cancer was confirmed via histopathology or cytology by a board-certified pathologist (JA, LD).

### Case selection for monitoring of MRD

The study on dogs with a clinical follow-up to study MRD was performed from October 2017 to October 2018. Dogs were prospectively enrolled in the study if they were diagnosed by histopathology with diffuse large B-cell lymphoma (DLBCL) and if they had started a chemotherapy protocol. Clinical staging was performed based on clinical examination, CT scan, and fine-needle aspiration for the detection of liver, spleen, and thorax involvement and bone marrow aspiration cytology for evaluating bone marrow infiltration. Evaluation of the response to treatment and relapse was performed at each visit according to the criteria described for multicentric lymphoma by VCOG (Veterinary Cooperative Oncology Group)^52^ based on clinical examination, with lymph node measurement and lymph node aspiration cytology performed at the discretion of the clinician. Plasma was sampled before the administration of chemotherapy every week during the first month, and subsequently every 2–4 weeks until relapse or death occurred.

### DNA extraction

Tumor DNA was extracted from RNAlater- or FFPE-preserved tumor samples using the DNA Nucleospin Tissue or FFPE Tissue DNA kits (MACHEREY NAGEL, Düren, Germany) according to the manufacturer’s protocol. cfDNA was extracted from 2 mL of plasma with NucleoSnap DNA Plasma (MACHEREY NAGEL, Düren, Germany), and eluted in 50 µL of elution buffer according to the manufacturer’s protocol. When less than 2 mL of plasma was available, the volume was made equivalent using PBS (Thermo Fisher Scientific, Waltham, MA, USA).

### PARR analysis

For dogs diagnosed with high-grade multicentric lymphoma, paired samples (tumor DNA and plasma DNA) were analyzed by PCR for antigen receptor rearrangement (PARR). M13-tailed primers were used to target the immunoglobulin heavy chain genes in B lymphocytes and the T-cell receptor γ in T lymphocytes (Table 2)^18,53,54^. Amplification was performed using the Type-it Multiplex PCR Master Mix (QIAGEN, Hilden, Germany), as described previously^55^ using the following conditions: denaturation at 92 °C for 5 min, 35 cycles of 95 °C for 8 s, 49 °C for 10 s, and 72 °C for 15 s (for IgH major, IgH minor, and TCR Burnett), or using 30 cycles of 95 °C for 45 s, 48 °C for 30 s, and 72 °C for 15 s (for IgH Tamura, T Yagihara VA, and Vb)^55^. The PCR amplicons were sequenced using the 3130 ABI sequencer (Applied Biosystems), and clonal profiles were determined using the Genemapper Software.

**Table 2:** Primers used in the study.

Primer targeted region	Primer sequence	Purpose	
IgH major	cacgacgttgtaaaacgacCAGCCTGAGAGCCGAGGACAC	PARR	
	TGAGGAGACGGTGACCAGGGT		
IgH minor	cacgacgttgtaaaacgacCAGCCTGAGAGCCGAGGACAC	PARR	
	TGAGGACACAAAGAGTGAGG		
TCR	cacgacgttgtaaaacgacCGTGTACTACTGCGCTGCCTGG	PARR	
	TGTGCCAGGACCAAGCACTTTGTT		

Primer targeted region	MIQE context	Purpose	Elongation temperature (°C)
PTPN11 hot spot 1 (E76K)	GATTCAGAACACTGGTGATTACTATGACTTGTATGGAGGGGAAAAGTTTGCCACGTTGGCT[G/A]AGTTGGTCCAGTATTATATGGAACATCACGGACAATTAAAAGAGAAGAATGGAGATGTTAT	ddPCR: mutation detection of PTPN11 E76K	58
PTPN11 hot spot 2 (G503V)	GGTGTTGACTGCGACATTGATGTTCCCAAAACCATTCAGATGGTGCGGTCTCAGAGGTCAG[G/T]GATGGTCCAGACAGAAGCACAGTACCGATTCATTTATATGGCTGTCCAGCATTACATTGAA	ddPCR: mutation detection of PTPN11 G503V	58
TRPM7	CATTACCTGTGTTTACTCCTCCAGTTAAAATCCAGGCTCCGGTTGTAACTGCAGCTTTAATAAGACCCTTTCCAAGCAGCTGCTTGATTCGTGGGTGAAGCTCAAATTTCTGCATGCCTCCAT	ddPCR: Copy Number determination	58
MdM2	TGATCTTCTAGGAGATTTGTTTGGAGTGCCAAGCTTCTCTGTGAAAGAGCACAGGTAAATTCTTCAGTTTAGTCCGTTGTAAAAAGCCAGCTGGGCAAACATTTCAGTTTACCTCCTCCTTTT	ddPCR: Copy Number determination	58
chr9 control	GCAGTCACCCCATCATCGTCCCGGCAAGGAAGCTTGTGCACAAACGCCAGCATCTGGTACTTGGCCCTGATGAATTCTGACTTGATGGGGCTGGGCAGGGCGCATAGGATGCGACGGTGAGCA	ddPCR: Copy Number determination	58
Specific probe for dog 1	F: CGGCtgTcTATTACTGTGCg	ddPCR MRD follow-up	60
	R: CTGCACCAGGACAAACAGAA		
	56-FAM/TGTCACCCA/ZEN/ATTGGAGCTGATCCC		
Specific probe for dog 2	F: ACTACCTCCGTGCTTTTGGT	ddPCR MRD follow-up	60
	R: CGTGTCCTCGGCTCTCAG		
	56-FAM/CGGCCGTAT/ZEN/ATTATTGTGTGAAGGC		
Specific probe for dog 3	F: ACACGGCCGTCTATTACTGT	ddPCR MRD follow-up	60
	R: GTTCCTGGCCCCAATAGTCA		
	56-FAM/ACGGCCGTC/ZEN/TATTACTGTTTCGGG		
Specific probe for dog 4	F: CGACGGTAGCTACGGTAACAAT	ddPCR MRD follow-up	60
	R: GGTCTCACCCGCACAGTAAT		
	56-FAM/AGACCGACG/ZEN/GTAGCTACGGTAACA		
chr26 control	F: TGAGACAACCCTCCC	ddPCR MRD follow-up	60
	R: CTTCTCTCGCTGAGAAAG		
	5HEX/CTGCCCGCT/ZEN/CTTTGATCCTGAGTC		

### Design of primers for digital droplet PCR (ddPCR)

ddPCR assays were designed using the Bio-Rad Droplet Digital PCR Assays and tools available online for mutation (https://www.bio-rad.com/digital-assays/#/assays-create/mutation) or copy number determination (https://www.bio-rad.com/digital-assays/#/assays-create/cnd) to detect the *PTPN11* mutations (E76K and G503V) or determine *MDM2, TRPM7,* and CFA 9 copy numbers, respectively. The region of CFA 9 was used as an internal control, as it was previously shown to have a high copy number stability in OMM^28,56^. The Bio-Rad MIQE Context locations of primers and probes are shown in Table 2.

For each dog with multicentric lymphoma examined for MRD, a PARR assay was performed on the tumor DNA as described above to isolate the clonal antigen receptor rearrangement. The PCR product was sequenced using a 3130 ABI sequencer with the BigDye Terminator v3.1 Cycle Sequencing Kit (Applied Biosystems, Foster City, CA, USA). The sequences overlapping the CDR3 (complementarity-determining region 3) were used to design clone-specific primers and FAM fluorescent probes for ddPCR analysis using the PrimerQuest Tool (Integrated DNA Technologies, Coralville, IA, USA). A region of CFA26 was used as the control gene, as it was previously found to be relatively stable in canine lymphomas^57^ (Table 2).

### ddPCR protocol and analysis

The ddPCRs assay was performed as previously described by Lodrini et al.^58^ or Mochizuki et al.^46^. Briefly, a 20 μL reaction volume containing the droplet Supermix (Bio-Rad Laboratories, Hercules, CA, USA) with a final concentration of 500 nM of forward and reverse primers, 250 nM of VIC- and FAM-labeled probes, and 8 µL of eluted cfDNA as input (mean: 15.2 ng) or 80 ng of DNA from tumor tissue isolated from each patient sample. Next, the PCR reaction mixtures were partitioned into an emulsion of approximately 20,000 droplets using a QX200 ddPCR droplet generation system (Bio-Rad). PCR was conducted using the GeneAmp PCR system 9700 thermocycler (Applied Biosystems) using the following program: 95 °C for 10 min; 40 cycles of 94 °C for 30 s, and an elongation temperature of 60 s, and subsequently 98 °C for 10 min. After PCR, the droplets were analyzed on a QX200 Droplet Reader (Bio-Rad). The concentrations of the target sequences were calculated using the Poisson distribution in the software Version 1.7.4 (Bio-Rad). Wild-type control DNA and the non-template control reactions were included in each experiment. To ensure experiment quality, wells with total droplet counts less than 8000 were considered invalid, and the experiment was repeated to obtain a sufficient number of droplets.

The *PTPN11* mutation frequency in cfDNA was determined by calculating the fraction of the mutant *PTPN11* molecule concentration A (copies/μL) relative to the wt *PTN11* reference molecule concentration B (copies/μL) (fraction = A/(A + B)). The estimation of the false-positive rate was determined by performing three experiments for each assay using the wt samples, in which the total numbers of detected mutated positive droplets were used to determine the thresholds above which the positive droplets in patient samples were considered as true positives. According to the input quantity, only reactions containing at least one, two, three, and four positive droplets for 0–7 ng, 7–15 ng, 15–100 ng and > 100 ng of input, respectively, were classified as positive in the ddPCR analysis.

In canine OMM, *MDM2* and *TRPM7*, copy numbers were determined using the QuantaSoft analysis software (version 1.7.4, Bio-Rad), which calculated the ratio of the target molecule concentration A (copies/μL) to the reference molecule concentration B (copies/μL) multiplied by the number of reference copies in the canine genome (copy number = (A/B)*2). These genes were considered amplified when the copy number was ≥ 3 and the confidence interval was > 2. The same method was used to quantify MRD in the plasma of dogs with lymphoma (A: lymphoma-specific antigen receptor rearrangement, and B: CFA 26 region, control, and ctDNA% = (A/B) × 100).

### Statistical analysis

Statistical analyses were performed using R studio software (Version 1.1.463; Vienna, Austria)^59,60^. The normal distribution of the data was evaluated using the Shapiro–Wilk test. When the data were not normally distributed, non-parametric tests were performed. The Wilcoxon test was used to compare the mean cfDNA concentrations and WHO stage in lymphoma and melanoma cases. A Kruskal–Wallis test was used to compare the cfDNA concentration in plasma between the different clinical groups. The Fisher exact or Chi-square tests were performed to evaluate associations between the presence of *PTPN11* mutations and clinical parameters. Finally, Spearman correlation tests were used to evaluate the correlation between lymph node size and ctDNA fraction in dogs with multicentric lymphoma that were receiving chemotherapy.

## Supplementary Information

Supplementary Information 1.

Supplementary Information 2.

Supplementary Information 3.

## Data Availability

The data from the article is available in the supplementary material provided with the article.
