# Supplementary material for: Circulating tumor DNA is detectable in canine histiocytic sarcoma, oral malignant melanoma, and multicentric lymphoma
Source: Sci Rep. 2021 Jan 13;11:877. doi: 10.1038/s41598-020-80332-y (PMC7806858; doi:10.1038/s41598-020-80332-y)
Supplement: Supplementary file 1 — Supplementary Information 1. [file 41598_2020_80332_MOESM1_ESM.docx]

**Circulating tumor DNA is detectable in canine histiocytic sarcoma, oral malignant melanoma and multicentric lymphoma**

Anaïs Prouteau, Jérôme Alexandre Denis, Pauline De Fornel, Edouard Cadieu, Thomas Derrien, Camille Kergal, Nadine Botherel, Ronan Ulvé, Mélanie Rault, Amira Bouzidi, Romain François, Laetitia Dorso, Alexandra Lespagnol, Patrick Devauchelle, Jérôme Abadie, Catherine André and Benoît Hédan*

Supplementary File 1. Clinical and molecular data of 4 dogs with DLBCL treated by chemotherapy.

Dog 1: This dog had a stage IV multicentric lymphoma and presented 17% of ctDNA in plasma before initiation of chemotherapy. A complete response (CR) was achieved 14 days after initiation of chemotherapy while molecular remission measured by ddPCR (ie. no ctDNA detected) occurred at day 33. At day 117 ctDNA was detected again in the plasma in a very small proportion (0.77%). The clinical relapse was objectified at day 159, ie 42 days after ctDNA detection. After reinduction, a second complete response was attained with a matched molecular remission. At day 274, the owner decided to stop chemotherapy. During disease course, ctDNA quantity and lymph node size were significantly positively correlated (Spearman correlation test, coefficient= 0.53, p=0.028).

Dog 2: This dog had a stage V multicentric lymphoma and we quantified 22% of ctDNA in its plasma before initiation of chemotherapy. The dog had only a partial response (PR) to therapy and was euthanized at day 50 after initiation because of disease progression (PD). Molecular monitoring fitted clinical observation as ctDNA was detected all the time during disease course. Lymph node size and ctDNA quantity were positively and significantly correlated (Spearman correlation test, coefficient= 0.88, p=0.03).

Dog 3: This dog had a stage IV multicentric lymphoma and presented only 6.25% of ctDNA in plasma before chemotherapy initiation. This lower ctDNA fraction may be due to a prednisolone treatment that was started 7 days before the beginning of the CHOP chemotherapy protocol. The dog experienced a CR at day 7 that matched with molecular remission. ctDNA was detected again at day 43 whereas the dogs was still in CR, but was not detected at the next sampling (day 63). The owner decided to stop chemotherapy at day 63 while the dog was in CR and molecular remission. One month after, the tumor recurred and a second chemotherapy was initiated but without significant response (stable disease –SD- followed by PD). This clinical evolution matched with the detection of high amount of ctDNA. The dog died one month after initiation of the second chemotherapy. Tumor burden and ctDNA quantity were significantly and positively correlated (Spearman correlation test, coefficient= 0.77, p=0.04).

Dog 4: This dog attained a CR at day 50 after chemotherapy initiation and the owner decided to stop the treatment at day 165 while the dog was still in CR. The complete clinical response was matched with a molecular remission that lasted until the dog was lost to follow-up. In that case the correlation between lymph node size and ctDNA quantity was positive but not significant (Spearman correlation test, coefficient= 0.48, p=0.18).
